# The home food environment and associations with dietary intake among adolescents presenting for a lifestyle modification intervention

**DOI:** 10.1186/s40795-018-0210-6

**Published:** 2018-02-06

**Authors:** Allison W. Watts, Susan I. Barr, Rhona M. Hanning, Chris Y. Lovato, Louise C. Mâsse

**Affiliations:** 1 School of Population and Public Health, University of British Columbia, 2206 East Mall, Vancouver, British Columbia V6T 1Z9 Canada; 2 Present address: Division of Epidemiology and Community Health, School of Public Health, University of Minnesota, Minneapolis, Minnesota 55401 USA; 3 Department of Food, Nutrition & Health, University of British Columbia, 2357 Main Mall, Vancouver, British Columbia V6T 1Z4 Canada; 4 School of Public Health and Health Systems, University of Waterloo, 200 University Avenue West, Waterloo, Ontario N2L 3G5 Canada; 5 School of Population and Public Health, University of British Columbia, 2206 East Mall, Vancouver, British Columbia V6T 1Z9 Canada; 6 School of Population and Public Health, University of British Columbia, 2206 East Mall, Vancouver, British Columbia V6T 1Z9 Canada; 7 Child and Family Research Institute, University of British Columbia, 4480 Oak Street, room F512a, Vancouver, BC V6H 3V4 Canada

**Keywords:** Home food environment, Adolescent overweight, Obesity treatment, Dietary intake

## Abstract

**Background:**

The home food environment may be an important target for addressing adolescent obesity. The aim of this study was to investigate associations between aspects of the home food environment and the diets of adolescents who present for obesity treatment.

**Methods:**

Cross-sectional baseline data were collected from 167 overweight/obese adolescent-parent pairs participating in an e-health lifestyle modification intervention. Adolescent intake of specific foods (fruit and vegetables, total fat, sugar-sweetened beverages, desserts/treats, and snacking occasions) was assessed by three 24-h dietary recalls, while household factors were collected from adolescent and parent questionnaires. Structural Equation Modeling, controlling for relevant covariates, was used to examine the relationship between adolescent diet and the following household factors: parent modeling, parenting style, family meal practices, and home food/beverage availability.

**Results:**

Findings reveal that few characteristics of the home food environment were associated with adolescent dietary intake. Greater home availability of high-fat foods was moderately associated with adolescent snack intake (β = 0.27, *p* < .001). Associations with fruit/vegetables and fat intake were small and some were in unexpected directions. Parent modeling of healthful food choices and healthier family meal practices were associated with lower availability of high-fat foods and treats in the home, but were not directly associated with adolescent diets.

**Conclusions:**

Parent modeling of healthy foods and positive mealtime routines might contribute to the healthfulness of foods offered in the homes of adolescents who are overweight/obese. Additional research is needed to better characterize the complex aspects of the household environment that influence adolescent diet.

**Electronic supplementary material:**

The online version of this article (10.1186/s40795-018-0210-6) contains supplementary material, which is available to authorized users.

## Background

A healthy diet during adolescence is important for optimal growth and for preventing the development of conditions such as diabetes, dental carries, and obesity [1]. Currently, adolescents consume too few fruits and vegetables and too many energy-dense nutrient-poor foods and beverages (e.g. sugary drinks, fast foods, and snacks) [2–5], and several studies report that these markers of poor diet quality are associated with obesity [6, 7]. Furthermore, one third of Canadian and American adolescents are overweight or obese [8, 9]; therefore, promoting a healthier diet is likely an important strategy for addressing childhood obesity. However, interventions have had limited success in changing adolescent dietary behavior, particularly in the context of obesity treatment programs [10].

In obesity treatment programs, parents are increasingly seen as important agents of behavior change because they are in control of broader aspects of the home, including the availability of foods and the rules that may support or hinder their children’s dietary choices [11]. Several models of the home food environment informed by social-cognitive and socio-ecological theories suggest that familial influences including parenting practices and other aspects of the home will shape the uptake of healthy dietary behaviors [12, 13]. Social aspects (e.g., parent role modeling, parenting style, mealtime routines, socio-demographic and economic characteristics), physical aspects (e.g, what foods and beverages are available and easily accessible) and the interplay between them have been associated with adolescent diet in previous studies [14–16]. This type of model has been tested in a population-based study examining adolescent fruit and vegetable (FV) intake. In particular, FV intake was influenced by availability in the home, and in turn, availability in the home was influenced by social support for healthful eating, family meal patterns, food security and socio-economic status (SES) [16].

There is limited evidence testing these home environment models for youth who are overweight or obese and seeking treatment. Some evidence comes from results of a parent-centered program focusing on promoting an authoritative parenting style, role modeling, and a healthier home food environment (e.g. availability, accessibility, meal routines), which found greater reductions in body mass index (BMI) than when children alone are targeted [17]. These findings have sparked increased interest in the role that parenting and home food environments may play for youth in obesity treatment programs. Further exploration of these influences on the diets of overweight/obese adolescents will inform intervention targets and help to improve the effectiveness of obesity treatment programs.

**Fig. 1:**
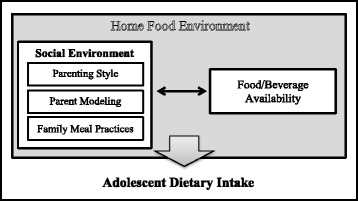
Proposed model of factors within the home food environment and their association with adolescent dietary intake. This conceptual model details the primary processes of interest, however, modeling will also take into account important covariates such as child age and sex, maternal education and household income

To build on the existing literature and gain insights that are directly relevant to improving adolescent obesity treatment programs, the aim of the present study was to test a structural equation model of associations between the home food environment and dietary intake among obesity treatment seeking adolescents. It was hypothesized that an authoritative parenting style, parent modeling of healthful food choices (FV and low-fat snacks), more healthful family meal practices (fewer meals in front of the TV and at fast food restaurants), reduced availability of less healthful foods and drinks (availability of select high-fat foods or non-diet soft drinks in their home) and high SES (higher education or income level) would be associated with more healthful dietary habits among overweight/obese adolescents. It was also hypothesized that social influences may indirectly influence adolescent dietary intake through associations with availability of less healthful foods in the home (Fig. 1).

## Methods

### Participants and procedures

This study utilizes baseline data collected from adolescent participants of an eight-month e-health obesity intervention, which included anthropometric measurements, questionnaires (Additional file 1), and three 24-h dietary recall assessments. In addition, one of their parents completed a baseline questionnaire on the home food environment. Participants were recruited from newspaper advertisements (62%), invitations sent to previous patients of a Children’s Hospital Endocrinology & Diabetes Unit (13%) and healthy weights clinic (15%), and other sources (e.g., word of mouth) (10%). Eligible adolescents were 11–16 years old and had BMI z-scores greater than one standard deviation from the mean, according to WHO age-and-gender matched growth charts [18]. Participants had to be residents of the greater Vancouver area with no plans to move within the study period, read at the grade 6 level and speak English. Exclusion criteria included comorbidities that required immediate medical attention, medical reasons that made physical activity too difficult, use of medication affecting body weight, diagnosis of Type 1 diabetes, or participation in another weight-loss program. Of the 183 parent-child pairs who completed the baseline assessment, seven did not meet eligibility requirements (e.g. BMI, reading level), three did not complete any 24-h dietary recalls, and six parents did not complete the home environment questionnaire yielding a sample of 167 parent-adolescent pairs for the present analyses. Written consent was obtained from all participants and this study was approved by the University of British Columbia and the University of Waterloo ethics boards.

### Measures

#### Outcome variable

Dietary Intake was assessed using a previously validated [19], computer-based 24-h dietary recall program employing a three-pass technique where participants were asked to report all foods/beverages that they consumed the previous day at breakfast, morning snack, lunch, afternoon snack, dinner, and evening snack. Over 900 brand or generic food items were available and participants were instructed to substitute foods not found (20% of recalls had at least one food item substituted). Photographs depicting measured portion sizes helped to estimate portion sizes and prompts allowed for the selection of toppings commonly eaten with certain foods (e.g. spreads on toast). A summary screen allowed participants to confirm or delete their selections. Dietary data were downloaded from the web survey and processed with The Food Processor software package (version 8.0, ESHA Research, Salem, OR, 2002) that uses the 2007 Canadian Nutrient File data (http://www.hc-sc.gc.ca/fn-an/nutrition/fiche-nutri-data/index-eng.php) to calculate nutrient and Canadian food group estimates.

Of the 167 adolescents examined in the present study, 76 provided all three days of dietary recalls, while 46 provided two days and 45 provided only one day. No differences by number of dietary recalls completed were found except for consumption of desserts/treats, which was significantly greater among those who completed more days of dietary recall (data not shown). Because few differences were found, dietary intakes were averaged across all available recalls to obtain daily estimates of: 1) servings of FV, 2) percentage of energy from total fat (Fat), 3) servings of sugar-sweetened beverages (SSB), 4) servings of desserts or treats (Desserts/treats), and 5) percentage of energy from snacking occasions (Snacks). Desserts/treats included food items commonly consumed for dessert or as a treat (e.g. cookies, cake, candy, chocolate, ice cream and chips), which are typically energy dense yet nutrient poor. Servings of SSB and desserts/treats were dichotomized (any vs. none) because they had a highly left-skewed distribution.

#### Independent variables

Parent Modeling was assessed with five items from the adolescent questionnaire: 1) My parents eat vegetables when I am with them; 2) My parents eat fruits when I am with them; 3) My parents eat salad at a restaurant when I am with them; 4) My parents eat low-fat snacks when I am with them; 5) My parents eat low-fat dressings with salads when I am with them. Responses to each item were coded on a 4-point scale (Never, Sometimes, Frequently, Always). These items were adapted from Cullen’s 15-item parent modeling scale [20], which also included additional items specific to particular meal times. Similar items have also been used to predict diet outcomes in adolescent samples [21].

Parenting Style was assessed with eleven items from the parent questionnaire such as wanting to hear about my child’s problems, knowing where my child is after school, and telling my child that I like him/her just the way he/she is. Responses to each item were coded on a 4-point scale (Never, Sometimes, Often, Always). These items were derived from Cullen’s 11-item authoritative parenting scale [22].

Family Meal Practices was assessed with seven items drawn from the Family Nutrition and Physical Activity Screening Tool [23], which was completed by parents: 1) eating breakfast together, 2) eating at fast food restaurants, 3) eating while watching television, 4) eating fruits and vegetables with meals or as snacks, 5) using pre-packaged foods for meals, 6) eating dessert regularly after dinner, and 7) eating dessert regularly in the evening. Responses were coded on a 4-point scale so that a higher score indicated more healthful meal practices.

Home Food Availability was assessed with eight items from the parent questionnaire. Participants were asked if the following seven food types were available in the past week (yes/no) and if they were low-fat (yes/no): 1) cookies, pies, cakes or snack cakes; 2) chips (e.g. potato, corn, tortilla or Doritos chips); 3) ice cream or frozen yogurt; 4) granola bars; 5) bacon/sausage; 6) hot dogs; and 7) frozen dinners. Similar to previous studies that summed food items into the total number of core foods versus non-core foods available in the home or the number of energy-dense snack foods [24, 25], availability items were split into two indices and summed to generate: 1) Availability of high-fat foods (bacon/sausage, hot dogs, frozen dinners; range = 0–3), and 2) Availability of high-fat treats (cookies/pies/cakes/snack cakes, chips, ice cream/frozen yogurt, and granola bars; range = 0–4). Items identified as low-fat versions were omitted. Availability of soft drinks was assessed with the following item: “Did you have regular sodas or soft drinks in your home in the past week?” These items were derived from a list of 15 items used in the Girls Health Enrichment Multisite Study [26, 27]. Similar items have been used to predict dietary intake in adolescent samples [21].

#### Covariates

Adolescent Age and Gender, Parent Ethnicity, Maternal Education and Household Income were based on parent self-report. Highest degree, certificate, or diploma of mother was obtained and responses were grouped into three categories: 1) Less than or equal to high school education; 2) Trade certificate, diploma, non-university certificate, or university certificate below a bachelor level; and 3) University degree or greater. Total income, before taxes and deductions, of all household members from all sources in the past 12 months was obtained and responses were collapsed into four categories: 1) ≤ $40,000; 2) $40,001–$80,000; 3) $80,001–$120,000; and 4) ≥ $120,000. Body Mass Index z-scores, based on sex and age, were computed from measured height and weight using the WHO method for children and adolescents (5–19 years old) [18].

### Analysis

Confirmatory Factor Analysis (CFA) was performed to determine if scale factor structures were supported in this sample. Availability of high-fat foods was conceptualized as an index and availability of soft drinks was assessed by only one item; therefore, they were not examined using CFA. Model fit was assessed using commonly accepted fit indices: Chi-square goodness of fit test (*p*-value ≥.15), Comparative Fit Index (CFI > .95), Root Mean Square Error of Approximation (RMSEA<.06 with an upper CI ≤ .08 and a p-value > .05), and the Standardized Root Mean Square Residual (SRMR<.08) [28]. Since the chi-square test is highly influenced by model complexity and sample size, and CFI and SRMR are highly influenced by the inclusion of non-significant paths, the RMSEA was the main index used to determine model fit [28]. A single model was built with all three latent constructs and the Maximum Likelihood Estimator was used. Internal consistency of items in each scale was determined by computing Cronbach’s alpha.

After the measurement models were refined, two structural equation models tested the conceptual model linking the home food environment to adolescent dietary outcomes: FV, Fat, SSB, Desserts/treats, and Snacks. For the analyses, servings of FV were expressed per 1000 kcal (to account for energy intake and to maintain a scale comparable with the other dietary variables). First, all of the independent variables were regressed on each dietary outcome to determine direct effects. Second, the independent variables were regressed on dietary outcomes as well as on home availability variables. Covariates included adolescent age, sex, maternal education and household income. The Means- and Variance- adjusted Weighted Least Squares (WLSMV) method of estimation was used to handle a combination of continuous and dichotomous outcome variables. WLSMV has been proposed as the best estimator when categorical data are present [29], was designed specifically for use with small and moderate sample sizes, and is fairly robust to non-normality [30, 31]. Model fit was assessed using the indices described earlier as well as the Weighted Root Mean Square Residual (WRMR). When using the WLSMV estimator, the RMSEA and WRMR are the best indices of model fit, with a WRMR of less than 1.0 and a RMSEA of less than 0.6 suggesting a good fit [28].

Missing data were handled using pairwise deletion (< 5% missing). All conceptual paths were included in the model and were considered significant at *p*-value< 0.05. All statistical analyses were conducted using MPlus® (version 7, Los Angeles, CA).

## Results

### Sample characteristics

The average age of adolescents was 13 and slightly more females participated than boys. Families were fairly evenly distributed across household income categories, while twice as many mothers had a university degree as compared to a high school degree or less. Families reported having more high-fat treats in the house than other high-fat foods and just over one third reported having non-diet soft drinks in the house (Table 1).

**Table 1:** Adolescent and household characteristics

	*N*	Mean	SD	Range	*n* (%)
Demographic Characteristics					
Age	167	13.2	1.8	11.0–16.0	
Sex (Female)	167				89 (53.3)
Body Mass Index (BMI zscore)^a^	167	2.7	0.9	1.1–6.7	
Weight (kg)	167	83.5	22.9	48.0–175.8	
Height (m)	167	1.63	0.1	1.4–2.0	
Maternal Education	167				
≤ High School Trade Certificate/Diploma ≥ University Degree					32 (19.2) 64 (38.3) 71 (42.5)
Household Income	167				
≤ $40,000 $40,001–$80,000 $80,001–120,000 ≥ $120,000					33 (19.8) 54 (32.2) 45 (27.0) 35 (21.0)
Parent Ethnicity (White)	165				77 (46.7)
Home Food Environment					
Availability of High-Fat Foods (0–3)	167	0.6	0.7	0–3	
Availability of High-Fat Treats (0–4)	167	1.9	1.2	0–4	
Availability of Soft Drinks (yes)	167				61 (36.5)
Authoritative Parenting (1–4)	159	3.5	0.5	2.1–4.0	
Parent Modeling (1–4)	162	2.5	0.6	1.0–4.0	
Family Meal Practices (1–4)	154	2.8	0.7	1.0–4.0	
Dietary Intake					
Fruit & Vegetables, servings/d	167	3.4	2.0	0.0–8.8	
Fat, % kcal/d	167	32.8	8.1	3.4–56.7	
SSB, consumed (yes)	167				88 (52.7)
Desserts/Treats, consumed (yes)	167				104 (62.3)
Snacks, % kcal/d	167	17.3	11.5	0.0–67.7	

*SD* standard deviation, *SSB* sugar-sweetened beverages, *BMI* Body Mass Index

^a^Based on WHO growth charts

### Measurement model

Initial results did not support the original factor structure of the data; however, after examination of modification indices, several post-hoc modifications with conceptual relevance were made to produce a measurement model that demonstrated good model fit. Retained items and fit indices are presented in Table 2.

**Table 2:** Measurement model of parenting constructs using confirmatory factor analysis

	Standardized Factor Loading^a^	Standard Error	Cronbach’s alpha
Authoritative Parenting			0.81
Listens to child’s problems	0.45	0.07	
Aware of where child is going	0.56	0.06	
Tells child when doing a good job	0.65	0.06	
Checks child’s homework	0.63	0.06	
Knows what child does with friends	0.72	0.05	
Likes child the way they are	0.60	0.06	
Tells child when to come home	0.75	0.05	
Parent Modeling			0.76
Parents eat fruits around child	0.50	0.07	
Parents eat salad at restaurants around child	0.60	0.06	
Parents eat low fat snacks around child	0.75	0.05	
Parents eat low fat dressings around child	0.82	0.05	
Family Meal Practices			0.60
Family eats fast food	0.71	0.09	
Family eats while watching television	0.42	0.09	
Family uses pre-packaged meals	0.66	0.09	
Family eats dessert after dinner	0.41	0.09	

Initial model fit: χ^2^(df = 249) = 494, p < .001; RMSEA = .08 [.07–.09], p < .001; CFI = .78; and SRMR = .09

Final model fit: χ^2^(df = 87) = 123, *p* < .01; RMSEA = .05 [.03–.07], *p* = .50; CFI = .94; and SRMR = .06

^a^Standardized factor loadings of final model, all significant at *p* < .001

Correlations between factors were as follows: 0.15 between authoritative parenting and parent modeling; 0.16 between authoritative parenting and family meal practices; and 0.25 between parent modeling and family meal practices

### Structural equation model

**Fig. 2:**
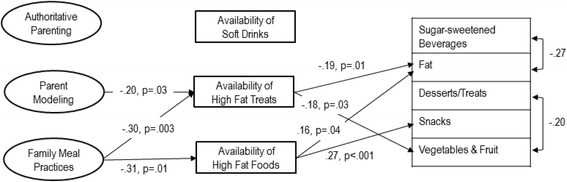
Structural equation model of factors within the home environment associated with the dietary intake of 167 overweight/obese adolescents. This figure presents only the significant standardized regression coefficients (which can be interpreted as correlations) and the full solution is presented in Table 3. These effects are corrected for the following covariates: child age and sex, maternal education, and household income. Non-significant paths are not shown for clarity

First, a model of direct effects was fit: χ^2^ (df = 252) = 352, *p* < .001; RMSEA = .05 [.04–.06], *p* = .56; CFI = .73; and WRMR = 0.98. No direct associations were seen with authoritative parenting, parent modeling, or family meal practices and dietary outcomes (data not shown). Second, a model with the addition of variables regressed on home food and beverage availability was fit: χ^2^ (df = 252) = 339, p < .001; RMSEA = .05 [.03–.06], *p* = .72; CFI = .76; and WRMR = 0.90 (see significant standardized coefficients in Fig. 2 and full solution in Table 3). The findings revealed that social variables (authoritative parenting, parent modeling, and family meal practices) had no direct effect on dietary outcomes; however, several social variables had a direct association with the availability of food and beverages in the home, which in turn, had a direct effect on dietary outcomes (Fig. 2). In both models, the CFI and χ^2^ *p*-value were not within suggested ranges, but the RMSEA and WRMR were. Examination of the modification indices did not uncover ways to improve the model and deletion of non-significant paths was not considered, given the confirmatory nature of our analyses.

**Table 3:** All estimated paths of the structural equation model examining direct and indirect effects (*n* = 167)

	Dietary Outcomes				
	Fruit & Vegetables	Fat	SSB	Desserts/Treats	Snacks
Home Food Environment	Standardized regression coefficient, *p*-value				
Availability of High-Fat Foods	−.145, *p* = .09	**.157,** ***p*** **= .04**	.190, *p* = .06	.159, *p* = .10	**.243**, ***p*** **< .001**
Availability of High-Fat Treats	**−.178,** ***p*** **= .03**	**−.186,** ***p*** **= .01**	.174, *p* = .10	.119, *p* = .28	−.018, *p* = .82
Availability of Soft Drinks	−.127, *p* = .12	−.060, *p* = .42	.154, *p* = .12	.142, *p* = .16	.003, *p* = .97
Authoritative Parenting	−.112, *p* = .15	.060, *p* = .36	.045, *p* = .63	−.019, *p* = .86	.061, *p* = .44
Parent Modeling	.068, *p* = .50	−.030, *p* = .75	−.039, *p* = .76	−.176, *p* = .20	−.046, *p* = .67
Family Meal Practices	−.190, *p* = .08	−.063, *p* = .53	.071, *p* = .59	.033, p = .82	−.159, *p* = .13
Covariates					
Maternal Education	.100, *p* = .24	**−.281, p < .001**	.007, *p* = .94	.036, *p* = .74	−.006, *p* = .94
Household Income	−.147, *p* = .08	**.176, p = .03**	−.004, p = .97	.075, *p* = .48	.001, *p* = .99
Age	−.116, *p* = .16	.087, *p* = .29	.016, *p* = .87	.012, *p* = .91	.026, *p* = .75
Sex (male)	−.065, *p* = .44	.031, *p* = .68	**−.205, p = .04**	−.110, *p* = .27	−.146, *p* = .09
	Home Availability Outcomes				
	High-Fat Food	High-Fat Treats	Soft Drinks		
Home Food Environment	Standardized regression coefficient, *p*-value				
Authoritative Parenting	.062, *p* = .41	.014, *p* = .85	.011, *p* = .61		
Parent Modeling	−.143, *p* = .10	**−.198**, *p* = .03	−.321, *p* = .31		
Family Meal Practices	**−.283**, *p* = .01	**−.286**, *p* = .01	−.054, *p* = .61		
Covariates					
Maternal Education	**−.204,** ***p*** **= .003**	−.028, *p* = .72	.010, *p* = .90		
Household Income	**−.165,** ***p*** **= .048**	−.134, *p* = .10	−.124, *p* = .40		

*SSB* sugar-sweetened beverages

Bolded values are significant at *p* < 0.05

A small number of variables were associated with adolescent dietary outcomes (Fig. 2). As hypothesized, availability of high-fat foods was associated with a greater percentage of energy from fat and from snacks. Greater availability of high-fat treats was associated with lower FV and unexpectedly with lower fat intake. Despite hypothesized associations, no relationships were found for Desserts/Treats and SSB intake or with the availability of soft drinks. Among demographic and socio-economic factors, adolescents from families with higher maternal education and with a lower income consumed lower percentage of energy from fat. In addition, males had lower odds of reporting SSB consumption (Table 3). Note that analyses with the percentage of energy from saturated fat versus total fat yielded similar results.

Some hypothesized relationships between factors in the social environment and the physical environment of the home were observed (Fig. 2). Healthful parent modeling and more healthful family meal practices were indirectly associated with dietary outcomes through home food availability. Adolescents who reported that their parents modeled healthful food consumption had fewer high-fat treats in their homes. Similarly, families reporting healthier family meal practices also reported reduced availability of high-fat foods and high-fat treats. Families with a higher maternal education and higher household income had lower high-fat food availability.

## Discussion

Few studies have examined the home food environment among adolescents with overweight/obesity. Families of adolescents who present for obesity treatment may provide valuable insights about what home environment characteristics need to be addressed to improve the effectiveness of these interventions. Results suggest that limited aspects of the home food environment are associated with the diets of treatment seeking adolescents; however, many expected associations were not found. In particular, home availability of non-diet soft drinks was not associated with decreased consumption of less healthful foods or beverages. In addition, more positive parent modeling and family meal practices were not directly associated with any dietary outcomes, but were associated with reduced availability of certain less healthful foods in the home. Mixed findings suggest that interventions that target both aspects of the social and physical environment of the home may help to support dietary intake among adolescents who are overweight/obese, but that they may be limited. Individual preferences and influences outside the home including peers, and community and school environments are likely shaping the diets of adolescents who are overweight/obese.

The strongest associations found in this study were between social aspects of the home food environment (modeling and meal routines) and having less healthful foods in the home. This finding is not surprising as parent preferences likely impact food purchases and have been found to predict the foods served to younger children [32]. In addition, families with meal routines such as consuming fast food meals more frequently have been found to report having chips and soft drinks available in the home and a higher intake of fast food and salty snacks by adolescents [33]. In contrast, we did not find a direct relationship with adolescent dietary intake, but social influences may indirectly shape what foods are made available or broader aspects of the home environment. For example, parents of overweight/obese adolescents who model healthful eating and create healthier meal routines may be more actively engaged in promoting healthful eating as a whole and thus, also making changes to other aspects of the home eating environment that influence diet. Interventions aimed at improving the quality of foods made available in homes may benefit from also targeting parenting behaviors, such as modeling and family meal practices. In contrast to previous studies [34], we did not identify any associations with an authoritative parenting style. These null findings may be explained by our measure, which did not identify the typical typologies of parenting (authoritarian, authoritative, permissive and disengaged) resulting in overlap with authoritarian parenting styles.

Previous studies have found positive associations between availability and adolescent consumption of a variety of foods/beverages including FV [35], non-core foods [24], less healthful foods [36], energy-dense snacks [35], and soft drinks [37], and many similar associations were found in the present study. However, there were many null findings and some associations were in an unexpected direction. In light of the small number of food items that were assessed for availability, those measured may represent less healthful food items that are in the home along with healthier options or that may be in most households for special occasions only (e.g., parties, the weekend). Since these families had presented for an obesity treatment program, they may have made changes to the home environment (e.g., eliminating particular foods) after enrolling in the intervention, but prior to the baseline data collection that did not yet translate into dietary change (and obscuring longer term patterns). Therefore, results may not reflect families that have not yet contemplated making environmental or behavioral changes in response to their child’s weight [38].

It should also be noted that several associations were significant, but had small effect sizes (< 0.23 or < 5% of the variance explained) and may explain some of the inconsistent associations observed. Associations with small effect sizes tend to be less stable and these findings should be interpreted with caution. While it remains difficult to determine how many subjects should be included in a SEM analysis to yield enough power, our study was likely powered to detect moderate effects based on the findings from simulation studies [39]. Thus it would be useful to replicate these analyses in a larger sample to test the stability of these associations. Other limitations of this study include that families of adolescents who are overweight/obese and who present for treatment may be influenced by a more complex set of individual and psycho-social factors influencing food choices or may make changes to their environment in response to their own or their children’s weight. Thus, findings are most applicable to the families of adolescents who present for obesity treatment in urban or suburban settings. This study also utilized cross-sectional data; therefore, precludes causal inferences. Furthermore, measures were not validated in a sample of adolescents with overweight/obesity and their parents and self-reported parenting practices and diets are susceptible to social desirability bias, which may have influenced the results towards a null finding [40]. The measure for family meal practices had particularly low reliability and may highlight the difficulty in measuring the home food environment, particularly in unique samples. Improved measures for assessing the home food environments of adolescents are needed. Finally, only a select number of dietary outcomes were examined as indicators of diet quality. Our dietary database precluded the examination of added sugars, for example, which may be an important indicator of a suboptimal diet among adolescents.

## Conclusions

Despite confirmation of some hypothesized relationships in the present study, many dietary factors were not associated with aspects of the home food environment. However, parent modeling of healthy foods and positive mealtime routines were associated with the healthfulness of foods offered in the homes of adolescents who are overweight/obese. It remains a challenge to characterize both dietary intake and the complex aspects of the household environment that influence diet. The home environment and its influence on diet may be unique for overweight/obese adolescents; thus, future research is needed to identify important influences of diet among this understudied group. Future research should also consider the role of environments outside the home on adolescent dietary behaviors, such as the school food environment, and eating out with peers.

## Additional file

Additional file 1: Parent questionnaire items assessing demographic characteristics and the home food environment. (DOCX 375 kb)

## Abbreviations

BMI: Body Mass Index
CFA: Confirmatory Factor Analysis
CFI: Comparatie Fit Index
FV: Fruits and Vegetables
RMSEA: Root Mean Square Error of Approximation
SES: Socioeconomic Status
SRMR: Standardized Root Mean Square Residual
SSB: Sugar-sweetened Beverages
WHO: World Health Organization
WLSMV: Means- and Variance- adjusted Weighted Least Squares
WRMR: Weighted Root Mean Square Residual
