# Supplementary material for: The home food environment and associations with dietary intake among adolescents presenting for a lifestyle modification intervention
Source: BMC Nutr. 2018 Feb 6;4:3. doi: 10.1186/s40795-018-0210-6 (PMC7050879; doi:10.1186/s40795-018-0210-6)
Supplement: Supplementary file 1 — Parent questionnaire items assessing demographic characteristics and the home food environment. (DOCX 375 kb) [file 40795_2018_210_MOESM1_ESM.docx]

1. People living in Canada come from different cultural and racial backgrounds. Please read all the categories and **select all that apply**.

 Aboriginal decent (e.g., North American Indian, Métis or Inuit (Eskimo))

 White

 Chinese

 South Asian (e.g., East Indian, Pakistani, Sri Lankan, etc…)

 Black (e.g., African, Haitian, Jamaican, Somali, etc…)

 Filipino

 Latin American

 South East Asian (e.g., Vietnamese, Cambodian, Malaysian, Laotian, etc…)

 Arab

 West Asian (e.g., Iranian, Lebanese, Moroccan, Afghan, etc…)

 Korean

 Japanese

 Other

If you selected other above, please specify. Otherwise skip to the next question._________________

2. What is the highest degree, certificate, or diploma **you have** obtained?

 **High school or less** than high school education

 **Trade certificate or diploma** from a vocational school or apprenticeship training

 **Non-university certificate or diploma** from a community college, CEGEP, school of nursing,

 **University certificate below bachelor’s level**

 **Bachelor’s degree**

 University degree or certificate **above bachelor’s degree**

3. What is the highest degree, certificate, or diploma **your spouse or common-law partner** has obtained?

 **Not applicable** since I do not have a spouse or partner

 **High school or less** than high school education

 **Trade certificate or diploma** from a vocational school or apprenticeship training

 **Non-university certificate or diploma** from a community college, CEGEP, school of nursing,

 **University certificate below a bachelor’s level**

 **Bachelor’s degree**

 University degree or certificate **above a bachelor’s degree**

4. What is your **marital status**?

 Single

 Married

 Living common-law

 Widowed

 Separated

 Divorced

5. Would you consider your family to be a **single or dual income family**?

 single income family

 dual income family – both working full time

 dual income family – only one of us is working full time

 dual income family – both working part time

 Not working at the present time

6 What is your best estimate of the **TOTAL** income, before taxes and deductions, of all household members from all sources in the past 12 months?

 $0 – $20,000

 $20,001 – $30,000

 $30,001 – $40,000

 $40,001 – $50,000

 $50,001 – $60,000

 $60,001 – $70,000

 $70,001 – $80,000

 $80,001 – $90,000

 $90,001 – $100,000

 $100,001 – $120,000

 $120,001 – $140,000

 $140,001 – $160,000

 $160,001 – or above

**7.The following questions ask about the number of people in your household.  Please read each question carefully.  If the question does not apply to you, select 0.**

How many adults **18 years of age or older** live in your household?

 1 (yourself only)

 2

 3

 4

 5

 6 or more

How many children between **12 and 17 years of age** live in your household?

 0

 1

 2

 3

 4

 5

 6 or more

How many children between **5 and 11 years of age** live in your household?

 0

 1

 2

 3

 4

 5

 6 or more

How many children **4 years of age or younger** live in your household?

 0

 1

 2

 3

 4

 5

 6 or more

**The next questions will ask about food availability in your home.**

1. Did you have **bacon or sausage** in your home in the **PAST WEEK**?

 No

 Yes 🡪 If “yes” What type of bacon or sausage did you have in your home in the **PAST WEEK**?

 Regular

 Reduced fat

2. Did you have **cookies, pies, cakes, or snack cakes** in your home in the **PAST WEEK**?

 No

 Yes 🡪If “yes” What type of **cookies, pies, cakes, or snack cakes** did you have in your home in the **PAST WEEK**?

 Regular

 Low fat

1. Did you have **chips (e.g., potato, corn, tortilla or Doritos chips)** in your home in the **PAST WEEK**?

 No

 Yes 🡪 If “yes” what type of **chips (e.g., potato, corn, tortilla or Doritos chips)** did you have in your home in the **PAST WEEK**?

 Regular

 Reduced fat or baked

1. Did you have **ice cream or frozen yogurt** in your home in the **PAST WEEK**?

 No

 Yes 🡪 If “yes” What type of **ice cream/frozen yogurt** did you have in your home in the **PAST WEEK**?

 Regular

 Low fat

5. Did you have **granola bars** in your home in the **PAST WEEK**?

 No

 Yes 🡪 If “yes” What type of **granola bars** did you have in your home in the **PAST WEEK**?

 Regular

 Low fat or fat free

6. Did you have **TV dinners or frozen entrees** in your home in the **PAST WEEK**?

 No

 Yes 🡪 If “yes” What type of **TV dinners or frozen entrees** did you have in your home in the **PAST WEEK**?  Regular

 Reduced fat

7. Did you have **hot dogs** in your home in the **PAST WEEK**?

 No

 Yes 🡪 If “yes” What type of **hot dogs** did you have in your home in the **PAST WEEK**?

 Regular

 Reduced fat

8. Did you have **sodas or soft drinks** in your home in the **PAST WEEK**?

 No

 Yes 🡪If “yes” What type **sodas or soft drinks** did you have in your home in the **PAST WEEK**?

 Regular

 Reduced calories or sugar free

**Please indicate the extent to which you never, sometimes, often, or always do the following:**

|  | **Never** | **Sometimes** | **Often** | **Always** |
| --- | --- | --- | --- | --- |
| 1. I want to hear about my child’s problems |  |  |  |  |
| 1. I make sure my child tells me where he/she is going |  |  |  |  |
| 1. I usually know where my child is after school |  |  |  |  |
| 1. I tell my child when he/she does a good job on things |  |  |  |  |
| 1. I am interested in my child’s school work |  |  |  |  |
| 1. I check to see if my child does his/her homework |  |  |  |  |
| 1. I often ask my child what he/she does with friends |  |  |  |  |
| 1. I make my child feel better when he/she is upset |  |  |  |  |
| 1. I tell my child that I like my child just the way he/she is |  |  |  |  |
| 1. I am usually pleased with how my child behaves |  |  |  |  |
| 1. I tell my child times when he/she must come home |  |  |  |  |
| 1. It is hard for me to say ‘no’ to my child |  |  |  |  |
| 1. I am always telling my child what to do |  |  |  |  |
| 1. I make rules without asking my child what he/she thinks |  |  |  |  |
| 1. I forget the rules I make for my child |  |  |  |  |
| 1. I can be talked into things easily |  |  |  |  |

| **Now that we have asked about your friends, we will ask the same questions about your PARENTS.** | | | | |
| --- | --- | --- | --- | --- |
|  | **Never** | **Sometimes** | **Frequently** | **Always** |
| My parents **eat vegetables** when I am with them |  |  |  |  |
| My parents **eat fruits** when I am with them |  |  |  |  |
| My parents **eat salad** at a restaurant when I am with them |  |  |  |  |
| My parents **eat low fat snacks** when I am with them |  |  |  |  |
| My parents **eat low fat dressings** with salads when I am with them |  |  |  |  |
